# Cardiovascular disease in a cohort exposed to the 1940–45 Channel Islands occupation

**DOI:** 10.1186/1471-2458-8-303

**Published:** 2008-09-02

**Authors:** Rosemary F Head, Mark S Gilthorpe, Allyson Byrom, George TH Ellison

**Affiliations:** 1 St George's, University of London, Cranmer Terrace, London, SW17 0RE, UK; 2 Biostatistics Unit, Centre for Epidemiology and Biostatistics, University of Leeds, Leeds, LS2 9LN, UK; 3 Guernsey Board of Health, Le Vauquiedor, St Martins, Guernsey, GY4 6UU, Channel Islands; 4 London Metropolitan University Graduate School, 277-281 Holloway Road, London, N7 8HN, UK

## Abstract

**Background:**

To clarify the nature of the relationship between food deprivation/undernutrition during pre- and postnatal development and cardiovascular disease (CVD) in later life, this study examined the relationship between birth weight (as a marker of prenatal nutrition) and the incidence of hospital admissions for CVD from 1997–2005 amongst 873 Guernsey islanders (born in 1923–1937), 225 of whom had been exposed to food deprivation as children, adolescents or young adults (i.e. postnatal undernutrition) during the 1940–45 German occupation of the Channel Islands, and 648 of whom had left or been evacuated from the islands before the occupation began.

**Methods:**

Three sets of Cox regression models were used to investigate (A) the relationship between birth weight and CVD, (B) the relationship between postnatal exposure to the occupation and CVD and (C) any interaction between birth weight, postnatal exposure to the occupation and CVD. These models also tested for any interactions between birth weight and sex, and postnatal exposure to the occupation and parish of residence at birth (as a marker of parish residence during the occupation and related variation in the severity of food deprivation).

**Results:**

The first set of models (A) found no relationship between birth weight and CVD even after adjustment for potential confounders (hazard ratio (HR) per kg increase in birth weight: 1.12; 95% confidence intervals (CI): 0.70 – 1.78), and there was no significant interaction between birth weight and sex (p = 0.60). The second set of models (B) found a significant relationship between postnatal exposure to the occupation and CVD after adjustment for potential confounders (HR for exposed vs. unexposed group: 2.52; 95% CI: 1.54 – 4.13), as well as a significant interaction between postnatal exposure to the occupation and parish of residence at birth (p = 0.01), such that those born in urban parishes (where food deprivation was worst) had a greater HR for CVD than those born in rural parishes. The third model (C) found no interaction between birth weight and exposure to the occupation (p = 0.43).

**Conclusion:**

These findings suggest that the levels of postnatal undernutrition experienced by children, adolescents and young adults exposed to food deprivation during the 1940–45 occupation of the Channel Islands were a more important determinant of CVD in later life than the levels of prenatal undernutrition experienced *in utero *prior to the occupation.

## Background

It has long been acknowledged that adult risk factors for cardiovascular disease (CVD), such as obesity and smoking, do not adequately explain the incidence of CVD in later life [1,2]. Indeed, as early as 1977, Anders Forsdahl postulated that there might be an important link between living conditions during childhood and adolescence and heart disease in later life [3]. This link was subsequently explored by David Barker and colleagues at the University of Southampton, whose research increasingly focused on the relationship between birth weight and health in later life, using low birth weight as a marker of undernutrition and adverse circumstances *in utero *[4]. This research suggested that fetal life might be a particularly sensitive period of development during which physiological mechanisms might be 'programmed' (i.e. permanently altered) in such a way that they lead to an increased risk of CVD in later life [5,6]. However, other researchers have speculated that postnatal development may contain even more sensitive periods than prenatal development [7], with puberty being a particularly sensitive period [8]. Indeed, a growing number of researchers now believe that health in later life is the result of exposures which accumulate and/or interact with one another across the entire life course, rather than those that 'programme' future health during a limited number of 'critical periods' [9].

Since it is difficult, expensive and time consuming to undertake prospective studies examining the relationship between living conditions in early life and health in later life, retrospective analyses of cohorts exposed to food deprivation during two 'natural experiments' – the 1944–45 Dutch famine and the 1941–44 Leningrad siege – have proved invaluable for clarifying this relationship. Although these analyses have primarily focused on the relationship between prenatal exposure to food deprivation and health in later life, they include some that have examined the potential impact of postnatal undernutrition during childhood, adolescence or early adulthood. These analyses have found a higher incidence of breast cancer [10,11] – though not other cancers [10,12-14] – amongst cohorts exposed to the Dutch famine during childhood and adolescence, higher mortality from ischaemic heart disease and stroke amongst men exposed to the Leningrad siege at 9–15 years of age [8,15] and higher systolic blood pressure amongst those exposed to the Leningrad siege at 6–15 years of age [8].

The present study aimed to clarify the nature of the relationship between undernutrition during pre- and postnatal development and CVD in later life. This involved examining a cohort of Guernsey islanders (born between 1923 and 1937) for whom data on birth weight were available (as a marker of prenatal nutrition), some of whom had been exposed as children, adolescents or young adults to food deprivation (i.e. postnatal undernutrition) during the 1940–45 German occupation of the Channel Islands [16]. The occupation culminated in a 9 month siege (from 1944 to 1945) when the islands were cut off from mainland France by the Allied liberation of Normandy following the D-Day landings. During this period, the official ration for islanders fell to around 1000 kcal per day – below the threshold identified as critical by researchers studying cohorts exposed to the Dutch famine [17]. By this stage of the occupation very little off-ration food was available [18], although the poor and those living in urban areas are likely to have suffered most, since these groups had the least funds to purchase whatever food was still available through the black market, and would have had least access to off-ration agricultural produce [19]. However, the paucity of fuel, water and soap, together with a more general decline in both the quantity and quality of food available, meant that the 1944–45 siege affected all but the most privileged islanders.

Previous studies examining the short-term consequences of the 1940–45 occupation on the health of Channel Islanders have found little evidence of any impact on self-reported birth weight [20] or on infant and under-5 mortality rates [21]. However, children resident in the islands during the occupation displayed slower rates of growth in both height and weight [22], as well as delayed age at menarche [23]. Preliminary analyses exploring the impact of exposure to the occupation on health in later life found that middle-aged men born in the Channel Islands in 1939–1940, before the occupation began, had significantly higher systolic blood pressure and blood glucose levels than those born in 1945–1946, after the occupation ended [24,25]. It therefore appears that postnatal exposure to the occupation during childhood and adolescence may have had some short- and long-term effects on health. The analyses that follow therefore aim to compare the impact of birth weight and postnatal exposure to the occupation on CVD in later life.

## Methods

The cohort was based on 1673 live births delivered by a community midwife in the 10 parishes of Guernsey between February 1923 and August 1937. The midwife's records included data on birth weight, date and place of birth, sex of the baby and gestational age in weeks. These data were confidentially matched by hand to the Guernsey birth registers, which provided additional information on the occupation of the father and marital status of the mother, as well as confirmation of the parish of residence at birth. Paternal occupation at birth was classified as manual vs. non-manual and used as a marker of social class in the analyses which follow. It was also reclassified as 'agricultural' vs. 'non-agricultural' to explore the potential impact of paternal occupations with access to off-ration foodstuffs during the 1940–45 occupation. Likewise, parish of residence at birth was used as a marker of parish of residence during the occupation and was classified as 'urban' or 'rural' to reflect differences in the availability of off-ration foodstuffs in different parishes during the occupation [26,27].

The cohort was then confidentially matched, again by hand, to the 'occupation register' compiled by the German Army during the 1940–45 occupation, which comprised a complete list of all resident islanders. In this way it was possible to identify those cohort members who were resident in Guernsey during the occupation, and those who were not (i.e. those who had left the island or had been evacuated before the 1940–45 occupation began). Finally, cohort members were confidentially matched to the hospital episode statistics (HES) database at the island's only hospital. HES for 1997–2005 were then searched for any admissions for CVD events – defined as myocardial infarction, stroke or unstable angina using the ICD-10 coding system.

Cox regression models were used to assess whether birth weight and/or exposure to the 1944–45 occupation might be associated with CVD in later life. Birth weight, which had been measured to the nearest 1/4 lb, was converted to kilograms (kg) and used as a continuous variable. The 'occupation register' compiled by the German army was used to identify those cohort members who had been exposed to the 1940–45 occupation (i.e. those resident in Guernsey) and those who had not (i.e. those who had left or had been evacuated from the island). The analyses that follow therefore provide hazard ratios for CVD-related hospital admissions corresponding to a 1 kg increase in birth weight, and to exposure to the 1940–45 occupation.

**Figure 1:**
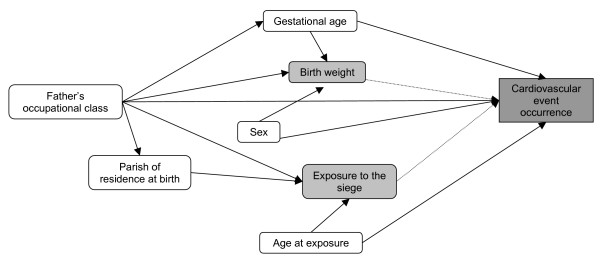
**Causal path diagram showing relationships between variables available for inclusion in the analyses**. Solid lines depict relationships presumed to be causal on the basis of previous studies or circumstances on Guernsey before, during and after the 1940–45 occupation. Dotted lines depict relationships examined by the analyses presented in Tables 1–3.

To inform the design of the Cox regression models, an analytical framework was drawn up in the form of a causal path diagram [28] which summarised the causal relationships considered likely to exist between all of the variables available for analysis (see Figure 1). Many of these relationships are reasonably straightforward and have been replicated in a number of previous studies. However, some of the relationships were particular to the present study and therefore had to be inferred. The latter included the relationships between paternal occupation at birth and parish of residence at birth, and between parish of residence at birth and CVD. For these relationships it was assumed that parish of residence at birth was associated with paternal occupation at birth [29] and would therefore be related to CVD in a similar way to paternal occupation at birth.

From Figure 1 it is evident that all but one of the covariates appeared to act as potential confounders because they preceded both of the exposures. The exception was exposure to the occupation, which would act as a competing exposure in analyses exploring the relationship between birth weight and CVD in later life, since it did not precede birth weight in this cohort and was not considered to be directly related to birth weight in the causal path diagram [20]. For this reason the analytical models exploring each of the exposure variables (birth weight and exposure to the occupation) included slightly different covariates acting as potential confounders and/or competing exposures. Three different sets of models were therefore developed to examine the relationships between each of these exposures and CVD in later life: one for birth weight, one for exposure to the occupation, and one exploring whether there was any interaction between birth weight and exposure to the occupation.

The first set of models (A: birth weight) included sex, gestational age at birth and paternal occupation at birth (as potential confounders) and exposure to the 1940–45 occupation (as a competing exposure). The first set of models also tested for any interaction between birth weight and sex. The second set of models (B: exposure to the occupation) included gestational age at birth, birth weight, paternal occupation at birth and parish of residence at birth (as potential confounders). The second set of models also tested for any interaction between exposure to the occupation and parish of residence at birth. Finally, the third model (C: birth weight × exposure to the occupation interaction) included sex, gestational age at birth, paternal occupation at birth and parish of residence at birth (as potential confounders) and tested for any interaction between birth weight and exposure to the occupation. All of the temporal variables available for inclusion in the analyses (such as date of birth and date of admission to hospital) were found to be correlated with one another. For this reason, the temporal variable most strongly related to CVD in later life (age at first admission to hospital) was included as the core temporal variable in the Cox regression analyses.

Sensitivity analyses were conducted to assess the possibility of selection bias occurring due to selective loss to follow-up in the HES database. These analyses assumed that those lost to follow-up did not appear in the HES database because they had *not *experienced a CVD event. Meanwhile, although the sample size used in the analyses that follow was predetermined by the numbers of cohort members who could be matched to the HES database, it was possible to conduct power calculations based on the cumulative incidence of CVD over a 7-year period estimated from the literature, and these were then compared with the actual effect sizes found in the Cox regression models.

Formal ethical clearance for the study was granted by the States of Guernsey Board of Health's research ethics committee.

## Results

### Loss to follow-up and selection bias

**Figure 2:**
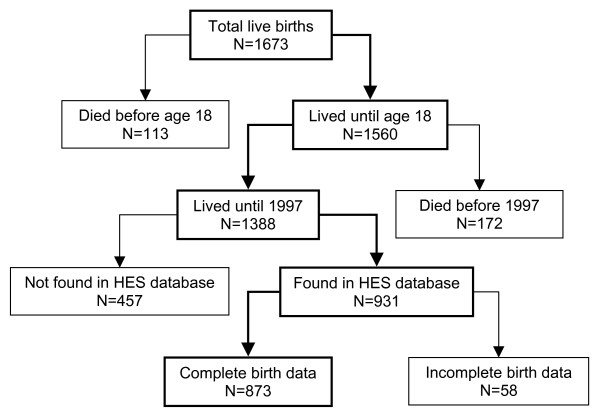
**Structure of the Guernsey midwife cohort**. Boxes in bold are those remaining in the cohort at each stage, and unbolded boxes are those removed from the cohort at each stage.

Of the original 1673 live births included in the cohort, 113 (6.8%) had died before the age of 18, and were therefore excluded from these analyses of CVD in adults. Data on hospital admissions in the HES database were identified for 931 of the remaining 1560 cohort members (see Figure 2). Of the 629 cohort members not found in the HES database, 172 were recorded in the Guernsey death registers as having died prior to the introduction of the HES database in 1997, leaving 457 cohort members unaccounted for and lost to follow-up. Some of these cohort members may have left Guernsey permanently before or after the 1940–45 occupation, while others may be residents who have yet to be admitted to the island's hospital. Of the 931 cohort members found in the HES database, 873 also had complete birth data and were included in the analyses that follow. This represents 52% follow-up of the original cohort and 63% follow-up of those who were alive when the HES database was first introduced. The matching process identified 225 individuals who were registered as being resident in Guernsey during the 1940–45 occupation, and these were therefore classified as exposed. Since the 'occupation register' used for this purpose provided a complete list of all islanders who were resident at the time of the 1940–45 occupation, none of the remaining cohort members (N = 648) would have been exposed to the occupation, and these were therefore classified as unexposed.

#### A: Birth weight and CVD

The first set of models (A: birth weight) showed that although there was some evidence of a positive relationship between birth weight and CVD, this was not statistically significant either before or after adjustment for sex, paternal occupation at birth, gestational age at birth and exposure to the 1940–45 occupation (see Table 1). To test for any interaction between birth weight and sex the data were analysed separately by sex. This comparison found that, although there was a modest inverse relationship between birth weight and CVD in men and a modest positive relationship between birth weight and CVD in women, a formal test for interaction between birth weight and sex was not statistically significant (p = 0.60). Meanwhile, paternal occupation at birth also approached statistical significance in its relationship with CVD, such that those cohort members whose fathers had manual occupations were more likely to have experienced CVD. However, exposure to the 1940–45 occupation was the only variable that displayed a statistically significant relationship with CVD, and this is examined in greater detail in the second set of models (B; see below).

**Table 1:** Cox regression analysis for models exploring the relationship between birth weight and CVD in later life (A).

	**Covariate ** (referent category)	**Hazard ** **Ratio**	**95% ** **confidence ** **intervals**	**p value**
**Unadjusted**	Birth weight (per kg increase)	1.30	0.82 – 2.07	0.26
**Unstratified, adjusted for potential confounders^1 ^and competing exposures^2^**	Birth weight (per kg increase)	1.12	0.70 – 1.78	0.65
	Female sex (male)	0.76	0.47 – 1.24	0.27
	Preterm gestational age (term)	0.26	0.04 – 1.91	0.19
	Manual paternal occupation (non-manual)	1.42	0.88 – 2.30	0.15
	Exposure to occupation (unexposed)	2.65	1.62 – 4.34	0.01
**Stratified by sex and adjusted for potential confounders^1 ^and competing exposures^2^**	Birth weight in men (per kg increase)	0.94	0.52 – 1.72	0.60^3^
	Birth weight in women (per kg increase)	1.60	0.73 – 3.50	

^1 ^Sex, gestational age at birth and paternal occupation at birth were all considered potential confounders. It is important to note that the interpretation of the estimated hazard ratios for potential confounders is not straightforward since the main exposure variable (birth weight) lies on the pathway between these potential confounders and the outcome variable (CVD in later life).

^2 ^Exposure to the 1940–45 occupation was considered a competing exposure.

^3 ^p value for interaction between birth weight and sex.

#### B: Exposure to the occupation and CVD

The second set of models (B: exposure to the occupation) confirmed that there was a significant relationship between exposure to the occupation and CVD, such that those who had remained on the island during the occupation had more than twice the HR of hospital admission for CVD in later life (see Table 2). After adjustment for potential confounders, this relationship remained statistically significant. Recoding paternal occupation at birth to test for any difference in CVD between cohort members whose fathers had agricultural vs. non-agricultural occupations (and would therefore have had more vs. less access to off-ration foodstuffs, respectively [18]), did not alter the relationship between this variable and CVD, nor did it affect the significant relationship between exposure to the occupation and CVD. However, parish of birth approached significance as a predictor of CVD in later life, and this is likely to reflect residual differences in social class related to the higher proportion of non-manual paternal occupations in urban parishes [28]. There was also evidence of a statistically significant interaction between exposure to the occupation and parish of residence at birth (p = 0.01), the size of the coefficient being higher amongst those resident in urban parishes (where the availability of off-ration food was lowest, but where paternal occupational class was highest [28]).

**Table 2:** Cox regression analysis for models exploring the relationship between exposure to the occupation and CVD in later life (B).

	**Covariate ** (referent category)	**Hazard ** **Ratio**	**95% ** **confidence** **intervals**	**p value**
**Unadjusted**	Exposure to occupation (unexposed)	2.88	1.78 – 4.66	0.01
**Unstratified, adjusted for potential confounders**^1^	Exposure to occupation (unexposed)	2.52	1.54 – 4.13	0.01
	Preterm gestational age (term)	0.28	0.04 – 2.05	0.21
	Birth weight (per kg increase)	1.15	0.73 – 1.83	0.55
	Manual paternal occupation (non-manual)	1.41	0.87 – 2.29	0.16
	Rural parish of birth (urban)	1.61	0.97 – 2.69	0.07
**Stratified by parish, and adjusted for potential confounders**^1^	Exposure to occupation in urban parishes (unexposed)	2.75	1.41 – 5.35	0.01^2^
	Exposure to occupation in rural parishes (unexposed)	2.28	1.08 – 4.81	

^1 ^Gestational age at birth, birth weight, paternal occupation at birth and parish of residence at birth were all considered potential confounders. It is important to note that the interpretation of the estimated hazard ratios for potential confounders is not straightforward since the main exposure variable (exposure to the occupation) lies on the pathway between these potential confounders and the outcome variable (CVD in later life).

^2 ^p value for interaction between birth weight and sex.

#### C: Interaction between birth weight and exposure to the occupation

The third model (C: interaction between birth weight and exposure to the occupation) divided the cohort into those who had been exposed to the occupation and those who had not and examined the relationship between birth weight and CVD in each of these groups to assess whether there was any evidence of an interaction (see Table 3). This found no evidence of an interaction between birth weight and exposure to the occupation, and a formal test for interaction was not statistically significant (p = 0.43).

**Table 3:** Cox regression analysis for model exploring interaction between birth weight and exposure to the occupation (C).

	**Covariate ** (referent category)	**Hazard ** **Ratio**	**95% ** **confidence ** **intervals**	**p value**
**Stratified by exposure to the occupation, and adjusted for potential confounders**^1^	**Exposed individuals**			
	Birth weight amongst those exposed to the occupation (per kg increase)	1.22	0.65 – 2.28	0.54
	Female sex (male)	0.63	0.33 – 1.17	0.14
	Preterm gestational age (term)	0.32	0.04 – 2.31	0.26
	Manual paternal occupation (non-manual)	0.93	0.49 – 1.77	0.82
	Rural parish of birth (urban)	1.93	0.99 – 3.75	0.05

	**Unexposed individuals**			
	Birth weight amongst those unexposed to the occupation (per kg increase)	0.92	0.44 – 1.90	0.81
	Female sex (male)	0.93	0.42 – 2.04	0.85
	Preterm gestational age (term)	-^2^	-^2^	-^2^
	Manual paternal occupation (non-manual)	2.53	1.15 – 5.58	0.02
	Rural parish of birth (urban)	1.15	0.51 – 2.58	0.73

^1 ^Sex, gestational age at birth, paternal occupation at birth and parish of residence at birth were all considered potential confounders. It is important to note that the interpretation of the estimated hazard ratios for potential confounders is not straightforward since the main exposure variable (birth weight) lies on the pathway between these potential confounders and the outcome variable (CVD in later life).

^2 ^There were no cohort members in this category who were preterm and who had also experienced a CVD event

### Sensitivity analyses and power calculations

The sensitivity analyses, which assumed that all those lost to follow-up had not been admitted to hospital for CVD, produced results that were no different for any of the three models in Tables 1, 2, 3. Meanwhile, the power calculations suggested that the number of cohort members with lower than mean birth weight in each of the analyses summarised in Table 1 (n = 425) was sufficient to detect just under a doubling of the hazard for CVD (or a HR of 1.92) at 90% power and a significance level of 0.05. If the Cox regression models in Table 1 had been calculated with 'lower than mean birth weight' as an exposure (rather than per kg increase in birth weight), the hazard ratio observed in the unadjusted model would have been 1.47. This is substantially smaller than that powered by the study, suggesting that the study was underpowered to examine a relationship of smaller magnitude between birth weight and CVD in later life and is therefore at risk of a type II error. For the models exploring the relationship between exposure to the occupation and CVD (Table 2), similar power calculations suggested that the number of cohort members who had been exposed to the occupation in the analyses summarised in Table 2 (n = 225) was sufficient to detect a doubling of the hazard for CVD (i.e. an HR of 1.99) at 90% power with a significance level of 0.05 and without adjustment for potential confounders. This is less than the actual effect size detected by either of the analyses presented in the second set of models (Table 2), and it therefore seems likely that the study was adequately powered for these models.

## Discussion

### Potential limitations

This study had a number of potential limitations which should be taken into account when interpreting its results. First, the cohort comprises only home births delivered by a single community midwife, and as such is unlikely to be representative of all births in Guernsey at that time. Indeed, when these births were compared to a 10% representative sample of births during the same period drawn from the Guernsey birth registers, it was found that the births delivered by the community midwife were significantly more likely to have taken place in urban parishes [29]. Sociodemographic differences were found in separate analyses comparing those who had been resident in Guernsey during the occupation and those who had been evacuated and subsequently returned [30], as well as analyses comparing cohort members of above and below average birth weight. However, because all the models adjusted for age (as this was the temporal variable included in the Cox regression analyses), parish of residence at birth (for models B), and sex (for models A), any potential selection bias related to these three sociodemographic variables should have been ameliorated in these analyses.

Nonetheless, the study suffers from substantial loss to follow-up, since the main analyses only include those cohort members who could be linked to the HES database. Those lost to follow-up would include any cohort members who had died prior to the introduction of the HES database in 1997, any who were evacuated from Guernsey and did not return, and any still living in Guernsey who had yet to attend the island's hospital and thereby enter the HES database (whether for CVD or non-CVD complaints). However, a comparison of cohort members included in the HES database with those who were not found few sociodemographic or clinical differences, although those included in the HES database were less likely to have died in Guernsey and more likely to have been exposed to the 1940–45 occupation. While such differences will affect the generalisability of the results, they may well be the result of population movement rather than pre-existing sociodemographic differences between those included in the analyses and those lost to follow-up. In this regard, the results of the sensitivity analyses were encouraging since these were not substantially different from the main analyses when all those lost to follow-up were assumed *not *to have experienced a CVD event.

Finally, the power calculations indicated that the analyses in the first set of models (A: birth weight) are likely to have been underpowered. If so, it is possible that a significant relationship may have been found between birth weight and CVD had a larger sample been available for analysis. However, because the modest relationship between birth weight and CVD amongst men and women combined was positive rather than inverse, and was only inverse amongst men when the analysis was stratified by sex, it seems unlikely that a larger sample size would have generated the statistically significant inverse relationship between birth weight and CVD that has commonly been observed in previous studies [4].

### Interpretation of findings

Notwithstanding these potential limitations, the lack of a significant inverse relationship between birth weight and CVD in this cohort runs contrary to the findings of numerous previous studies [6,31-33]. There was also no apparent interaction between birth weight and exposure to the occupation, suggesting that this had not masked any underlying association between birth weight and CVD. However, in many of the previous studies examining the relationships between birth weight, CVD and associated risk factors, these relationships were only apparent after adjusting for one or more measures of current body size – an analytical approach which can invoke a statistical artefact known as the 'reversal paradox' [34,35]. Although it was not possible to adjust for current body size in the present study, it would not have been appropriate to do so had data on current body size been available. Indeed, the absence of a significant negative relationship between birth weight and CVD in the present study adds to concerns that previous reports of such a relationship may be partly artefacts of the 'reversal paradox'.

Meanwhile, the results of both the first and second sets of models (see Tables 1 and 2) seem to provide clear evidence that exposure to the 1940–45 Channel Islands occupation was associated with an increased likelihood of hospital admission for CVD in later life. Differential exposure to undernutrition and related deprivation in early life between those who remained in Guernsey during the 1940–45 occupation and those who left or were evacuated before the occupation began [16,27] is one plausible explanation for these findings. Likewise, it is possible that the stress associated with the occupation may have had a number of permanent psychosocial effects that led to a differential risk of CVD in later life. However, those cohort members who were evacuated from the islands before the occupation began often left their families behind and were sent in large groups to unfamiliar towns in the UK, many of which experienced sustained bombing during the war. It therefore seems likely that both resident and evacuated islanders would have experienced some form of stress, whilst only those who remained on the island would have experienced the undernutrition and related deprivation associated with the 1940–45 occupation.

It is nonetheless possible that these findings reflect residual confounding for pre-existing sociodemographic differences between islanders resident in Guernsey throughout the 1940–45 occupation and those who left the island or were evacuated before the occupation began [30]. Given that the analyses presented here had limited access to sociodemographic variables (such as parish of residence and paternal occupation at birth), it seems likely that these analyses will have suffered from residual confounding. However, it is reassuring that the increase in CVD amongst cohort members resident on the island during the 1940–45 occupation remained statistically significant when paternal occupation was recoded as 'agricultural' vs. 'non-agricultural' and in the sensitivity analyses which assumed that cohort members missing from the HES database had *not *experienced CVD.

We are therefore confident that the results of these analyses strengthen the evidence provided by two previous studies of cohorts exposed in childhood to nutritional deprivation during the Leningrad siege, which found that cardiovascular mortality and systolic blood pressure were significantly elevated among those exposed at the age of 9–15 years [15], while men exposed at the age of 6–8 years also had increased mortality from ischaemic heart disease [8]. While these are similar findings to those observed in the present study, it is nonetheless important to note that conditions during the 1941–44 Leningrad siege were much more severe than those in Guernsey during the 1940–45 occupation and siege, with widespread starvation of the civilian population [36]. Nonetheless, one explanation for the Leningrad studies' findings was that children over the age of 12 appeared to have received disproportionately fewer rations than younger children, and a similar scenario has been described in accounts of the 1940–44 German occupation of Belgium [37] and in accounts of rationing from the 1940–45 occupation of the Channel Islands, which suggest that the young and the elderly suffered most [26,27,38]. Indeed, as in Leningrad, some accounts from the Channel Islands describe a scenario in which those in their early teenage years bore the brunt of inadequate food supplies during the occupation because those over 12 years old were often expected to work yet did not receive proportionately higher rations [39].

Moreover, there is further evidence that children and adolescents might have experienced longer-term developmental delays as a result of the Channel Islands siege, in the form of delayed age at menarche [23] and slower growth rates [22]. In particular, a study of Jersey schoolchildren during the occupation found that children who remained on the island displayed significantly slower growth compared to children in the UK [22]. While the lower rates of weight gain were temporary, the lower rates of height gain appeared longer lasting, indicating that exposure to the occupation may have had some longer-lasting developmental effects. Similar patterns of impaired growth were observed following the 1940–44 German occupation of Belgium, where girls who reached puberty during this time grew up to be shorter and lighter adults [37]. Likewise, in a study of women exposed to the Dutch famine, those exposed at the ages of 0–9 and 12–16 were found to have shorter stature as adults [40]. It is therefore possible that these effects on the stature of children and adolescents may reflect the developmental mechanism(s) through which exposure to the 1940–45 occupation of the Channel Islands might have led to an increased risk of CVD in later life.

This interpretation is supported by the significant interaction between exposure to the occupation and parish of residence at birth (used as a marker of parish of residence during the occupation), which indicated that exposure to the occupation was more strongly associated with CVD amongst those born/living in urban parishes where there was less access to off-ration foodstuffs [26,27]. Authors studying the immediate health effects of the Dutch famine have found similar differences between those living in rural and urban areas, and also attributed these to disparities in food availability therein [41]. As such, the statistically significant interaction between exposure to the occupation and parish of residence at birth observed in the present study supports the conclusion that the differences in hospital admission for CVD were due to undernutrition and related deprivation during the 1940–45 occupation. The interaction between exposure to the occupation and parish of residence at birth also raises the possibility of either a linear relationship or threshold effect involving the severity of food deprivation during the occupation and subsequent CVD.

## Conclusion

The present study aimed to explore the impact of undernutrition at various stages of early development on the subsequent risk of CVD in later life. In contrast to many previous studies, there was little evidence of an inverse relationship between birth weight (as a marker of nutrition *in utero*) and CVD in later life, although these analyses were substantially underpowered. In better powered analyses, there was clear evidence that exposure to nutritional deprivation during the 1940–45 occupation of the Channel Islands in childhood, adolescence and young adulthood was associated with an increased risk of CVD in later life. These findings suggest that, in this cohort, postnatal undernutrition associated with exposure to the 1940–45 occupation may be a more important determinant of CVD in later life than the levels of undernutrition experienced *in utero *prior to the occupation.

## Competing interests

The authors declare that they have no competing interests.

## Authors' contributions

RFH contributed to the design of the study, collection of data, analysis and interpretation, and also drafted and approved the manuscript. MSG contributed to the design of the study, analysis and interpretation, and also assisted with drafting the manuscript and approved the final version. AB contributed to the collection of data and approved the final manuscript. GTHE contributed to the conception and design of the study, collection of data, analysis and interpretation, and also assisted with drafting the manuscript and approved the final version.

## Pre-publication history

The pre-publication history for this paper can be accessed here:


